# Gate-to-Gate Life Cycle Study and Techno-Economic Analysis of an Industrial Process for Producing Densified Polystyrene from Recycled Expanded Polystyrene

**DOI:** 10.3390/polym18010034

**Published:** 2025-12-23

**Authors:** Eliana Berrio-Mesa, Alba N. Ardila A., Erasmo Arriola-Villaseñor, Santiago A. Bedoya-Betancur

**Affiliations:** Research Group on Environmental Catalysis and Renewable Energies (CAMER), Faculty of Sciences and Education, Politécnico Colombiano Jaime Isaza Cadavid, Medellín 050022, Colombia; eliana_berrio27121@elpoli.edu.co (E.B.-M.); erasmoarriola@elpoli.edu.co (E.A.-V.); santiago_bedoya27081@elpoli.edu.co (S.A.B.-B.)

**Keywords:** expanded polystyrene (EPS), circular economy, mass and energy balance, energy losses, financial feasibility, environmental analysis, carbon footprint, recyclable waste management

## Abstract

In this study, material and energy losses were systematically assessed, together with a comprehensive economic and environmental evaluation, for an industrial expanded polystyrene (EPS) recycling process implemented under a circular economy framework at a company located in Medellín, Colombia. The system boundaries were clearly defined, and detailed mass and energy balances were performed using operational data collected over a six-month period. The process achieved a yield of 78.09 percent in the production of densified polystyrene from post-consumer EPS, with the main material losses attributed to solid residues and water losses during processing. The total energy consumption was 7350.34 kWh, of which 55.46 percent corresponded to energy losses, predominantly thermal losses associated with the EPS melting stage. Techno-economic evaluation indicated that the process is financially viable over a twelve-year operational horizon. Furthermore, the environmental assessment demonstrated a 68.44 percent reduction in carbon footprint, underscoring the strong potential of this recycling route as a sustainable and effective alternative for the management of recyclable solid waste.

## 1. Introduction

The circular economy, applied to waste recycling, represents an important strategy for transforming waste into valuable resources, significantly contributing to reductions in environmental impacts such as soil degradation, depletion of natural resources, and global pollution [1,2,3]. This approach aims to extend the life cycle of materials by keeping their value within the economy for as long as possible [4]. In doing so, it minimizes waste generation and promotes the continuous reintegration of materials into the productive system [5]. The fundamental principles of this economic model are the reduction, reuse, recycling, and recovery of materials [6], prioritizing efficient resource use while fostering sustainable economic growth [7,8].

The production of plastic materials such as polyethylene terephthalate (PET), polystyrene (PS), polyurethanes (PURs), polyvinyl chloride (PVC), high-density polyethylene (PE), polypropylene (PP), resins, and fibers made from polyester, polyamide, and acrylic [9] have not been exempt from the environmental problems associated with the linear economy model, nor from the challenges posed by the transition toward the circularity of these materials [10]. A notable example is expanded polystyrene (EPS), one of the most widely used inert plastics globally due to its low cost. Composed of 95% polystyrene and 5% pentane gas [11], its high market value is due to its dimensional stability, resistance to impact, moisture, corrosion, and photolysis [12]. Additionally, its lightness, durability, low thermal conductivity, high acoustic absorption, and limited degradability make it a thermoplastic that is difficult to replace, with a wide range of applications [13,14,15,16,17,18,19,20].

In the packaging sector, EPS is extensively used to protect fragile products and preserve food. Moreover, it has applications in agriculture, the automotive industry, construction (as thermal and acoustic insulation), and in the manufacturing of sports equipment and protective helmets [21,22,23]. According to estimates from Grand View Research, the global EPS market is projected to reach USD 13.5 billion by 2028, with an annual growth rate of 4.8% between 2021 and 2028, driven mainly by the construction and packaging sectors [13,15]. However, once EPS enters a distribution chain and reaches the end of its useful life, most of this material lacks adequate recovery or recycling systems, leading a large portion of it to accumulate in landfills or dumps. Due to its low density and the large volumes of space it occupies, EPS can persist for hundreds of years, exacerbating solid waste management issues [6,17,18]. This improper final disposal not only affects linear economy models but also poses a challenge for circular economy systems, which offer viable solutions to reincorporate post-consumer EPS into the production chain efficiently and sustainably.

In this regard, various international EPS recycling technologies have been developed in recent decades, presenting different levels of technological maturity, energy requirements, and scalability [11,14,20]. Starting with mechanical recycling by extrusion/densification, which is mainly reported in Europe and other industrialized regions, post-consumer EPS is converted into pellets by melting and extrusion, making it the most commercially available technology [10,14]. Nevertheless, its performance depends heavily on the quality and purity of the input stream, as the presence of contaminants or thermal degradation of EPS affects the stability of the recycled product [10,11]. This dependence on clean streams limits its adoption in Latin American contexts, where heterogeneous post-consumer streams predominate [12].

On the other hand, solvent dissolution processes use organic solvents capable of collapsing the foam and recovering high-purity polystyrene [16]. Despite their effectiveness, these methods present challenges associated with solvent handling, recovery, operating costs, and regulatory requirements, which restrict their industrial deployment in countries with less developed chemical infrastructure [14,16]. Along the same lines, chemical and thermochemical recycling routes, such as pyrolysis, depolymerization, and catalytic technologies, have been extensively studied for their ability to process contaminated streams and recover monomers or fuels. Nonetheless, these techniques require significant investment, greater operational complexity, and stable markets for the products generated, which limits their implementation in scenarios where industrial scale and supply stability are not fully consolidated [14,20].

In contrast, the industrial process evaluated in this study, based on pretreatment, thermal compaction, and continuous extrusion, represents a mature technology with low technical complexity, aligned with the actual operating conditions in Latin America, where post-consumer flows from domestic and commercial sources predominate [12,17,18]. This comparison shows that, although there are more advanced international alternatives, the process analyzed is representative and suitable for middle-income countries, where viability depends on operational simplicity, stability of waste flow, and lower investment costs.

Similarly, the incorporation of the circular economy in the creation of new products from expanded polystyrene (EPS) has gained importance in recent years, focusing on extending the lifespan of the material through recycling and reuse. In this context, Life Cycle Assessment (LCA) has become an essential tool for evaluating the sustainability of these circular practices, enabling the identification of environmental impacts at all stages, from production to final disposal of recycled EPS [22].

LCA is essential for understanding whether efforts to reintegrate EPS into the production chain truly contribute to a significant reduction in carbon emissions, energy consumption, and waste generation [24,25]. Furthermore, it is important to conduct environmental impact assessments to measure the actual scope of the circular economy as applied to EPS, including aspects such as resource efficiency and the reduction in the carbon footprint compared to the traditional linear model of EPS [26,27]. Alongside this environmental analysis, it is also necessary to perform a Techno-Economic Analysis to determine whether the implementation of circular systems for EPS is economically viable in the long term [28]. This analysis should consider the operating costs associated with EPS recycling, investment in technology and equipment, and the financial benefits derived from the process, ensuring that the circular economy of EPS is not only sustainable from an ecological perspective but also financially feasible for a company.

For this reason, the present research aimed to develop a study and analysis of the industrial recycling process of expanded polystyrene under a circular economy approach, evaluating material flows, energy, carbon footprint, and water footprint. This analysis will include the identification of the environmental and economic impacts of the system, complemented by a techno-economic evaluation to determine the financial feasibility of the process.

## 2. Materials and Methods

In this study, a gate-to-gate Life Cycle Assessment (LCA), along with a Techno-Economic Analysis (TEA), was conducted to evaluate an industrial process for producing densified polystyrene from post-consumer expanded polystyrene (EPS). Based on the case study of a company in the city of Medellin, Colombia the densified polystyrene was obtained through a thermo-mechanical process, which operates at temperatures between 170 and 200 °C, in which the blowing agent, composed mainly of pentane and isopentane, is removed. During this process, the EPS, originally exhibiting a light and porous structure, is transformed into a compact form of high-density polystyrene, known as densified polystyrene. This resulting material has a hard, glassy texture with enhanced physical properties compared to the original EPS, making it suitable for industrial applications that require greater mechanical strength and durability.

### 2.1. Definition of System Scope and Boundaries

The study was conducted through visits to a production facility located in Medellín, Antioquia, which utilizes recycled expanded polystyrene (EPS) as a raw material for the generation of densified polystyrene. During these visits, process data were collected, enabling the definition of the study system, setting its boundaries, and delimiting the set of unit processes involved in the transformation of recycled EPS. These processes included the collection and pretreatment of the material, as well as the densification stages through thermal and mechanical processes, allowing for the characterization of material and energy flows to be subsequently evaluated in terms of carbon and water footprint and the economic feasibility of the system [29,30]. On the other hand, the functional unit defined for this study is 1 kg of recycled EPS obtained at the industrial plant evaluated. All material and energy flows and environmental loads are reported in relation to this unit.

The analysis was performance using a gate-to-gate approach, considering only the material and energy flows that occur within the operational boundaries of the recycling plant. The stages of virgin EPS production, transport to the plant, use phase of the recycled material, and end-of-life scenarios are outside the defined scope, as the objective of the study is to evaluate the direct environmental performance of the industrial process using primary operating data.

### 2.2. Gate-to-Gate Life Cycle Inventory (LCI)

Based on the establishment of gate-to-gate production system boundaries, a Life Cycle Inventory (LCI) Analysis was conducted to collect data for quantifying the inputs and outputs of the production system [31,32,33]. This stage of the study is essential, as it enabled the balancing of energy and material flows within the system under study.

**Figure 1:**
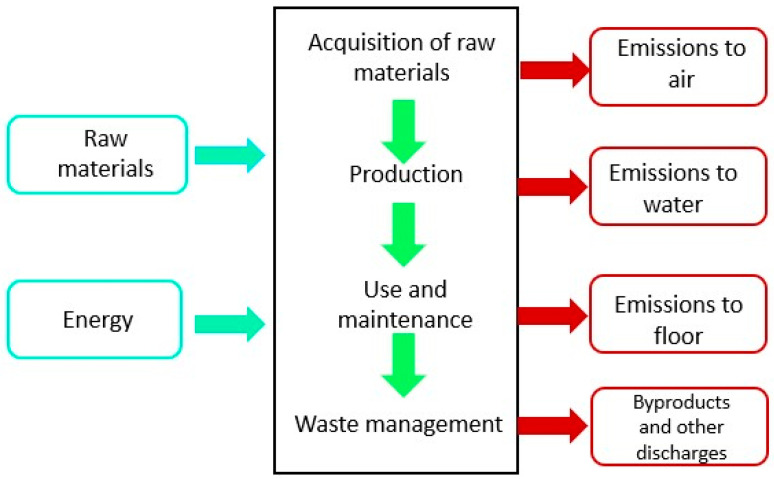
Life Cycle Inventory applied to a unit process within the system.

For the inputs, both material resources (recycled EPS and other waste types derived from EPS pretreatment) and energy consumption for each unit process (including thermal and mechanical energy) were quantified. Regarding the outputs, the analysis considered not only the final product (densified polystyrene) but also atmospheric emissions in terms of carbon footprint, water consumption and wastewater discharge, and solid waste that could potentially be released into the soil (Figure 1).

Based on the Inventory Analysis, a Sankey Diagram was developed to visually represent the flows of energy and materials throughout each unit process. This diagram enabled the identification of resource inputs and outputs at each stage of the system, facilitating the analysis of material and energy use efficiency, as well as losses within the system. This approach allowed for the evaluation of inefficiencies and improvement opportunities in both material and energy aspects. Additional data were obtained from the ECOINVENT database to supplement the analysis and were compared with those collected during visits to the study system.

On the other hand, a cut-off allocation strategy was applied, whereby recyclable by-products generated in the process (cardboard, metals, and minor plastic waste) are excluded from the inventory, as they represent less than 2% of the total mass flow and do not influence the environmental impact of the system. Therefore, the environmental impacts are assigned in their entirety to the main product, recycled EPS.

### 2.3. Life Cycle Environmental Impact Assessment

The ILCD 2011 Midpoint+ method was used for the Life Cycle Impact Assessment using the openLCA software (version 1.11.0), along with ELCD databases. These tools enabled the analysis of the environmental impacts of producing densified polystyrene from expanded polystyrene waste [33,34]. This comprehensive approach allowed for a detailed assessment of the environmental impacts associated with the production process and its life cycle.

It is important to highlight that all data entered into openLCA for applying the ILCD 2011 Midpoint+ method were sourced from the previously conducted mass and energy balance. Subsequently, the annual carbon footprint was calculated in terms of carbon dioxide emissions to the atmosphere. Moreover, the carbon footprint was determined by considering emission factors provided by the Mining and Energy Planning Unit (UPME) under Resolution No. 000320 of 2022 [34], which updates the emission factor for the National Interconnected System for 2021, used in greenhouse gas (GHG) emission inventories and mitigation projects for these gases [35]. It should be noted that the main carbon footprint value reported is obtained using ILCD 2011 Midpoint+, given its alignment with European LCA practices and its integration into openLCA. For comparative purposes, two additional values are included: IPCC 100-years and the national emission factor for the Colombian electricity system. The differences between them are explained by their methodological foundations. For example, ILCD incorporates multiple characterization mechanisms, IPCC evaluates only GWP100, and the national factor reflects exclusively the carbon intensity of the electricity consumed in Colombia. However, it is clarified that the main value is that reported with ILCD 2011 Midpoint+.

### 2.4. Technical–Economic Assessment (TEA)

In this study, the economic viability of producing densified polystyrene at an industrial scale from recycled EPS was evaluated, considering factors such as raw materials, electricity, labor, land, and equipment. The data used for this evaluation were provided by the producing company.

The associated costs were calculated, and the production value was estimated in Colombian pesos (COP), and subsequently expressed in US dollars (USD) according to the applicable exchange rate. To analyze financial viability, the Internal Rate of Return (IRR), Net Present Value (NPV), and Benefit–Cost Ratio (B/C) were calculated on an annual basis. This evaluation considered a 4.8% increase in costs due to inflation, an opportunity rate of 9%, and a loan covering 50% of the initial investment with an interest rate of 12%. Additionally, the consumer price index (CPI) and income taxes were established. Based on these analyses, annual costs and the initial investment required to start the company were determined, along with the estimation of annual revenues and financial viability over 8 and 12 years, represented through IRR, NPV, and the Benefit–Cost Ratio [28].

## 3. Results and Discussion

### 3.1. Process Description and System Boundaries

The process of obtaining densified polystyrene from post-consumer expanded polystyrene (EPS) consists of five unit processes, each playing a significant role in ensuring the efficiency and quality of the final product.

#### 3.1.1. Reception of Recycled Expanded Polystyrene (EPS)

**Figure 2:**
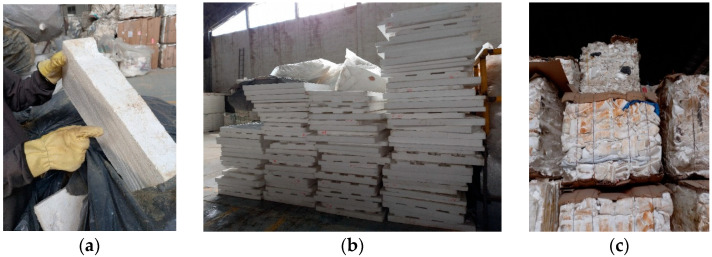
Reception of Recycled Expanded Polystyrene ((**a**). Packaging; (**b**). Sheets; (**c**). Compacted).

The recycled EPS comes from various post-consumer sources, such as the construction sector, packaging, and clean food containers. The material may arrive in different forms, such as sheets or compacted. At this stage, it is verified that the EPS is suitable for the recycling process and is classified according to its type (Figure 2). Proper classification of the EPS is crucial to facilitate the subsequent steps and ensure that the material meets the necessary requirements for further processing.

#### 3.1.2. Cleaning of Recycled Expanded Polystyrene (EPS)

This process is particularly important for material coming from the construction sector and compacted EPS. The primary objective of this cleaning phase is to remove contaminants or residues that could interfere with the process of obtaining densified polystyrene. Additionally, this phase allows for a more accurate assessment of the actual yield of the recycled material while protecting both the processing equipment and the personnel involved in the process.

The EPS used in construction tends to be contaminated with materials such as concrete and plastic waste, which must be removed manually. The presence of concrete is particularly problematic, as its hardness can damage the main equipment used in the melting of EPS. This cleaning process ensures the integrity of the machinery and guarantees a continuous and efficient flow in the subsequent stages of recycling. On the other hand, in the case of compacted EPS, contaminants tend to be more varied, including scrap, plastics, PET, cardboard, and straps (binding bands). These materials must also be removed manually, following strict safety protocols, as handling solid waste can pose risks to personnel. The use of appropriate personal protective equipment (PPE) is mandatory, in accordance with company regulations, to minimize any risk of accidents or exposure to hazardous substances.

Once the cleaning process is complete, two types of waste are generated: ordinary waste and recyclable waste. Ordinary waste, which cannot be recycled or reused, is managed by a company for external disposal. On the other hand, recyclable waste, such as plastics, PET, cardboard, scrap, and straps, is sorted and sold to other companies that use it as raw material for the manufacture of new products or for recycling purposes. Thus, it was determined that for every 28,110 kg of dirty recycled EPS, approximately 26,343 kg of clean recycled EPS are obtained, resulting in a yield of 93.71%, with the remaining 6.29% corresponding to ordinary waste (1.68%) and recyclable waste (4.60%).

#### 3.1.3. Melting and Degassing of Recycled Expanded Polystyrene

Once the reception and cleaning processes have been completed and the raw material meets the required characteristics, the degassing stage begins. In this process, pentane and isopentane are extracted from the expanded polystyrene (a polymer derived from styrene, obtained from naphtha, benzene, and ethylene) using specialized grinding and pressing machinery. This thermal-mechanical treatment is carried out at temperatures between 170 and 200 °C. As a result, high-density polystyrene is obtained in a compact, semi-solid form at temperatures above 100 °C, requiring immediate molding by the operators. Considering the yield from the previous stage of “Cleaning of Recycled EPS” along with the yield obtained in this degassing stage, it has been determined that from 28,110 kg of EPS entering the plant, 21,951.3 kg of high-density polystyrene are produced, representing a total yield of 78.09%. It should be noted that the EPS degassing stage takes place in an open system, without mechanisms for recovering, burning, or confining residual pentane. Given that there are no direct measurements of uncontrolled emissions and the study is based on a gate-to-gate approach, this flow was not included as an direct emission in the LCA model.

#### 3.1.4. Molding and Cooling of Densified Polystyrene Blocks

**Figure 3:**
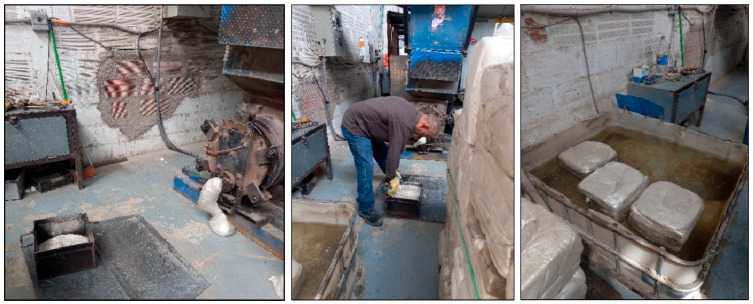
Molding and Cooling Stage of Densified Polystyrene.

In this phase of the process, the high-density polystyrene is shaped into blocks measuring approximately 40 cm × 35 cm. Layers of densified polystyrene are slowly added until the square mold is completely filled, and hardens simultaneously. The blocks are then removed from the mold and cooled in a water tank for approximately 10 to 20 min. Once the product has cooled to room temperature (Figure 3), it is weighed and subsequently transferred to the storage area for further commercialization.

#### 3.1.5. Storage and Marketing

Once cooled, the blocks of densified polystyrene are weighed using an industrial scale to ensure precise control of the weight of each block, which generally ranges from 8 to 11 kg. Subsequently, the blocks are organized into stacks. Before marketing, the blocks are carefully packaged to protect them from potential damage during transport. This process ensures that the material arrives in optimal condition to buyers, who are usually companies dedicated to plastic recycling or the manufacturing of new products from recycled materials.

### 3.2. Gate-to-Gate Life Cycle Inventory Analysis (LCI)

#### 3.2.1. Mass Balance

**Figure 4:**
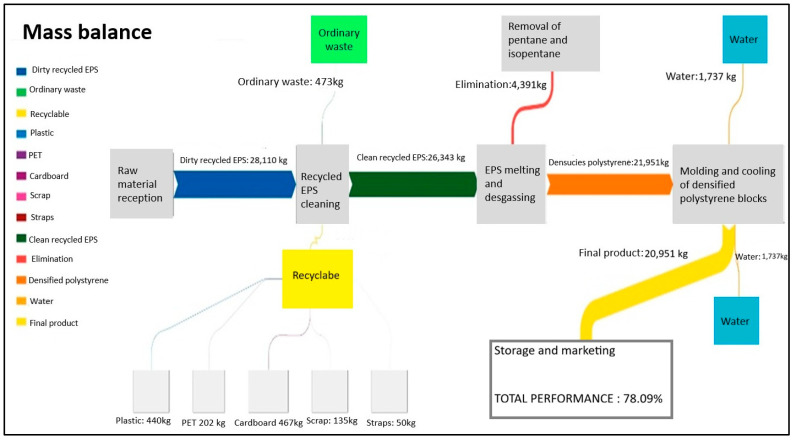
Sankey Diagram—Material Flow.

The Sankey flow diagram (Figure 4) is presented below, detailing the process for obtaining the blocks of densified polystyrene. This mass balance includes various aspects, such as production days, the amount of post-consumer EPS processed monthly and its associated costs, the ordinary waste generated, and the recyclable waste produced, such as plastics, PET, scraps, cardboard, and strapping bands. Additionally, the specific yields from the melting and degassing process of clean EPS are considered, which are essential for evaluating process efficiency [36,37].

According to the results obtained from the mass balance, which includes the inventory of material inputs and outputs in the study system, it was determined that for every 28,110 kg of recycled expanded polystyrene, 21,951.3 kg of high-density polystyrene are produced, resulting in a total yield of 78.09% (Figure 4). Material losses are primarily associated with ordinary waste generated during the cleaning process, which is managed and disposed of in landfills by a specialized waste management company. On the other hand, recyclable waste, such as plastics, PET, scrap metal, cardboard, and strapping bands, is valued and integrated into other production chains through commercialization, generating additional income for the company.

Additionally, water losses were identified in the system, particularly during the cooling process of the densified polystyrene blocks. Although the tempering tank for the finished product is filled only once a week, the water is subsequently discarded due to the murky color it acquires after several days of use, representing an additional loss in the process.

The performance results of the study system are comparable to the data reported in the literature for the recycling of post-consumer expanded polystyrene and its processing to obtain recycled polystyrene. Previous studies have reported yields ranging from 50.10% [6] to 85% in the pelletization processes of recycled EPS through mechanical methods [17]. However, the yield for obtaining a final product depends on the study system and the limits established to meet the objectives of the inventory analysis, particularly those related to material flows.

It is noteworthy that as more detailed data about the system are obtained, new variables can be identified that directly influence the performance of the process, allowing for continuous adjustments and improvements. This approach emphasizes the importance of constant monitoring and process adaptation to optimize resource use and minimize losses [38,39,40].

#### 3.2.2. Energy Balance

To assess energy consumption in the five operational units of the expanded polystyrene densification process, the following considerations were taken into account:

− Overall Energy Consumption for an average monthly input of 28,110 kg of recycled EPS into the system.
− Electrical energy consumption was primarily associated with the operation of five key pieces of equipment: a paddle mill, a cutter, the main melting unit, and two industrial scales.
− Human resources.
− Energy efficiency of electric motors and thermal machine efficiency.
− The energy assessment is based on the Colombian electricity matrix, which is characterized by a high share of hydroelectric generation (approximately 70% annually). For this reason, the impact associated with the energy consumption of the process is dominated by this source, in line with the geographical representativeness of the study. Although comparison with alternative generation technologies (solar, wind, thermal, among others) is methodologically relevant, their full incorporation exceeds the scope of the defined door-to-door life cycle analysis study.

**Figure 5:**
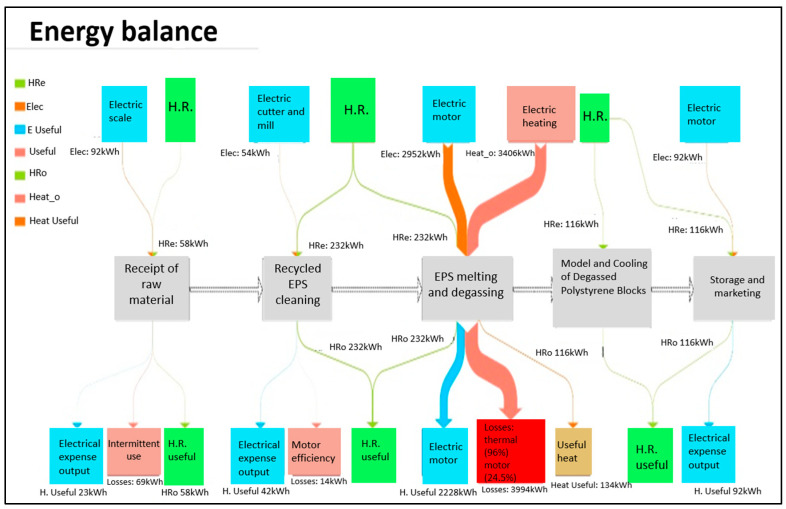
Sankey Diagram—Energy Flow. The different background colors used in the boxes of the energy balance diagram are intended to visually distinguish the nature of the process elements and energy flows involved. Gray boxes represent the main unit operations of the industrial process. Blue boxes correspond to electrical energy inputs associated with equipment and machinery (e.g., electric motors, electrical devices). Red/orange boxes indicate thermal energy inputs and thermal losses related to heating operations. Green boxes represent human resources (H.R.), indicating the contribution of manual labor required at different stages of the process. This color-coding scheme is applied consistently to improve readability and to facilitate interpretation of the energy balance.

As shown in the energy flow diagram of the Sankey type (Figure 5), the highest energy flows are concentrated in the melting and degassing stages, where both the use of electric motors and thermal energy converge [36,37]. These stages are the most energy-intensive due to the high heat required to melt and densify the expanded polystyrene.

Energy losses in the densification process of expanded polystyrene (EPS) were primarily identified in three operational units: Raw Material Reception, Cleaning of Recycled EPS, and EPS Melting and Degassing (Figure 5).

In the raw material reception stage, energy consumption was associated with the use of an industrial scale, which operates during the 26 working days of the process, remaining on for 8 h per day and consuming a total of 91.5 kWh. However, field observations revealed that this equipment is actually in use only for short periods, totaling a maximum of 2 h of real daily operation. By adjusting the calculation to the effective operating time, actual energy consumption was reduced to 22.88 kWh, representing a loss of approximately 68.64 kWh due to the time the equipment remains on without active use.

The cleaning of recycled EPS, which includes shredding and cutting stages using a blade mill and a cutter, also exhibited significant energy losses. An energy efficiency of 75.5% was considered for the motors, resulting in a useful output of 41.86 kWh and a loss of 13.6 kWh. These losses indicate unutilized energy during the operation of these mechanical devices, suggesting potential opportunities for enhancing operational efficiency by optimizing usage times and motor maintenance.

The EPS melting and degassing stage is the most energy-intensive phase. With a motor efficiency of 75.5%, losses of approximately 723.12 kWh were reported. These are compounded by thermal process losses, representing 96% of the total consumption of 3405.60 kWh, equating to an additional loss of 3268.88 kWh. Thus, total energy losses in this unit amount to 4106 kWh, establishing it as the primary source of inefficiency in the system.

A global analysis of the system revealed that processing 28,110 kg of recycled EPS requires a total of 7350.34 kWh of energy, of which only 3273.87 kWh (44.54%) are utilized as useful energy outputs, while 4076.48 kWh (55.46%) are lost. These figures underscore the need to optimize the stages of the process with higher energy consumption, particularly the melting and degassing phases. Comparing these results with the energy requirements of similar processes reported in the literature, specifically those documented in the database [41,42], reveals a high similarity in energy consumption. For instance, Zhang et al. (2021) reported an energy requirement of 6255.31 kWh for the production of foam boards with 10% recycled polystyrene content [43]. Although the processes for producing densified polystyrene and foam boards differ, both share intensive thermal energy use, highlighting the importance of understanding consumption ranges for processes involving recycled EPS.

The analysis of energy consumption and losses in the expanded polystyrene densification process provides critical insights into the efficiency of recycling systems and their environmental impact. Given that thermal processes represent the largest source of energy consumption and potential fossil fuel emissions [42], it is essential to adopt practices aimed at reducing these losses. For example, the incorporation of more efficient technologies and exploration of renewable energy alternatives could not only improve process efficiency but also enhance the sustainability of production chains within the framework of the circular economy.

### 3.3. Analysis of the Environmental Impact Assessment of the Life Cycle

**Figure 6:**
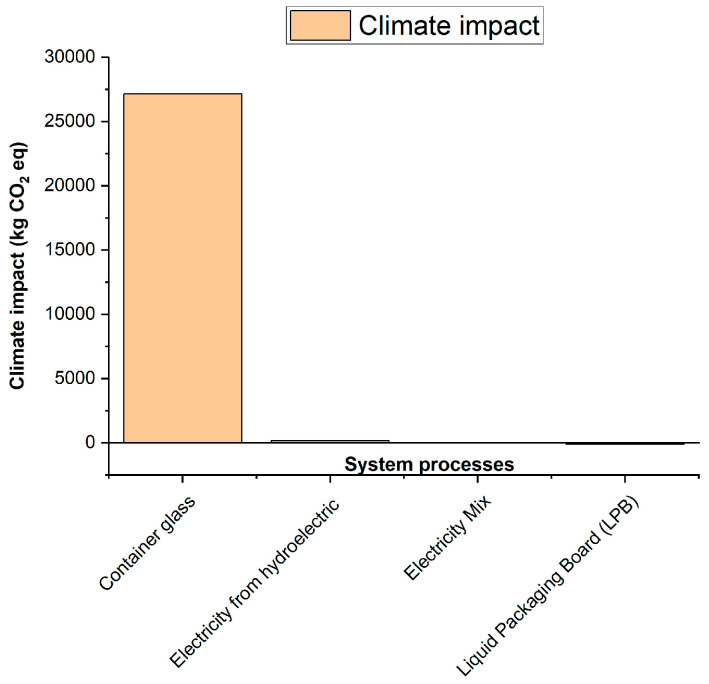
Contribution to Climate Change.

**Figure 7:**
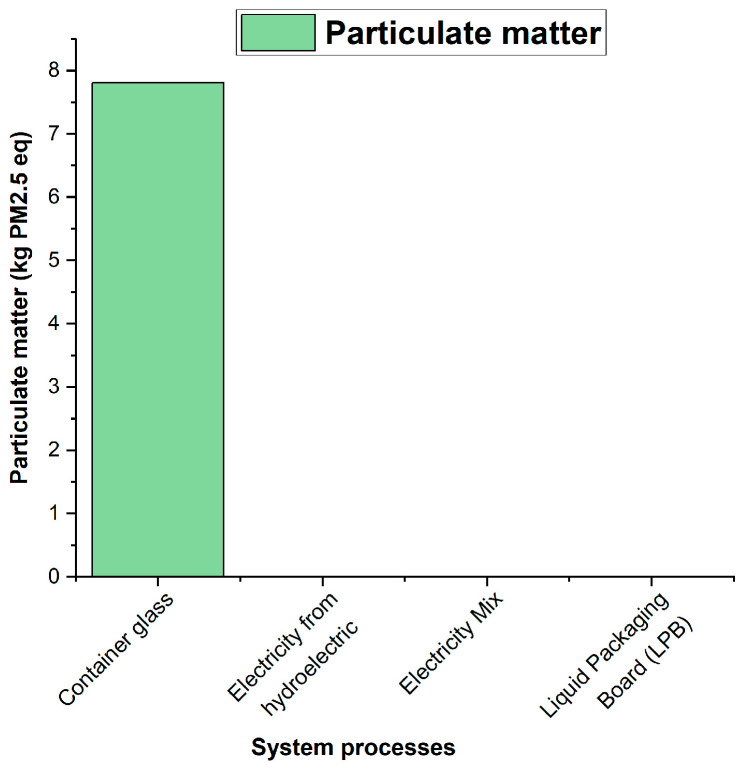
Contribution of Particulate Matter to the Atmosphere.

**Figure 8:**
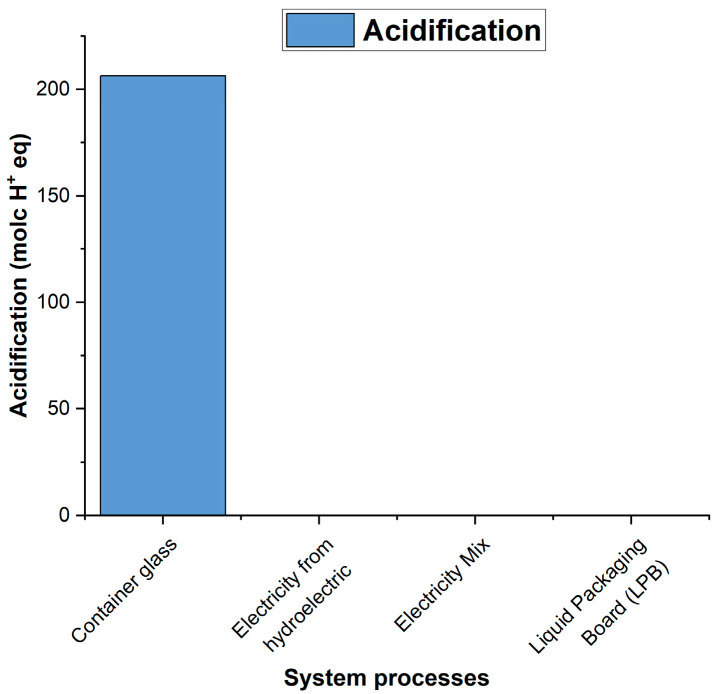
Contribution to Acidification.

**Figure 9:**
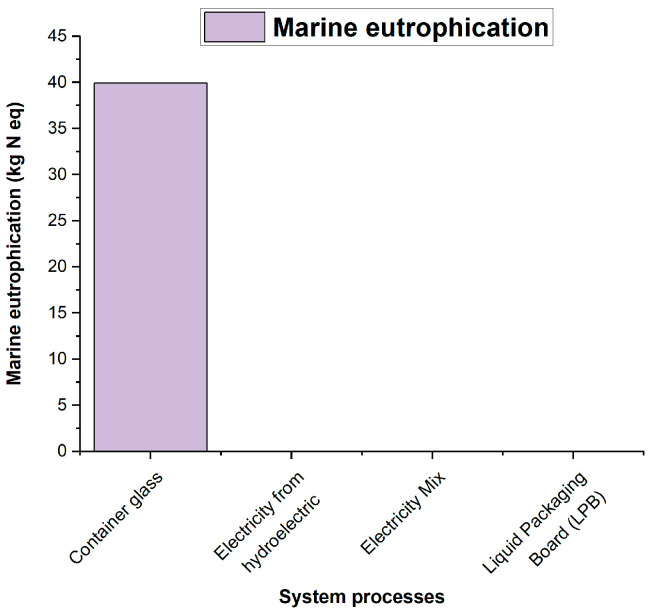
Contribution to Marine Eutrophication.

According to the results obtained using the ILCD 2011 Midpoint+ life cycle impact assessment method, the environmental impacts of the densified polystyrene production process are primarily observed in categories such as climate change (Figure 6), atmospheric particulate matter (Figure 7), acidification (Figure 8), and marine eutrophication (Figure 9). These impacts are directly associated with the systems and subsystems involved in hydroelectric power generation, a resource extensively used in this type of industrial process.

As can be seen in the figures above, the processes “Container glass,” “Electricity from hydroelectric power plants,” “Electricity Mix—GB,” and “Liquid Packaging Board (LPB)” appear among the main contributors to the impacts of climate change, particle formation, acidification, and marine eutrophication. It is important to note that these processes do not correspond to actual operations in the system analyzed; their presence is due to the background processes integrated into the ecoinvent database during the LCA calculation. In ecoinvent, generic flows associated with transport, energy consumption, or the use of materials modeled in reference processes are connected to supply chains that include, for example, glass or laminated cardboard, which explains their contribution in the impact analyses. This behavior is characteristic of multi-category methods such as ILCD, in which openLCA identifies and reports the background processes with the highest environmental impact within the entire network of interdependencies.

Hydropower has been a crucial pillar for social and economic development, providing reliable electricity. However, despite its advantages over fossil fuels, it is essential to recognize that it also generates significant environmental impacts. The perception of hydropower as a “clean” alternative often downplays the negative effects associated with its exploitation, which can be substantial depending on the type, scale, and location of hydroelectric facilities.

One of the primary impacts of this form of energy generation is the flooding of large land areas for reservoir creation, which results not only in the loss of local biodiversity but also in the displacement of communities near the affected zones [44]. Additionally, river ecosystems experience profound and irreversible changes, such as alterations to flora and fauna, disruption of local climate patterns, and substantial changes in water quality, flow, and availability. According to [45], these alterations not only impact natural habitats but also compromise long-term ecological stability, creating imbalances that may persist long after the construction of hydroelectric infrastructure.

Despite being a renewable energy source that emits fewer greenhouse gases than fossil fuel-based technologies, hydropower presents significant challenges regarding sustainability. These include high capital costs associated with its implementation and environmental impacts stemming from intensive natural resource use. Therefore, while hydropower has greatly contributed to reducing pollutant emissions compared to fossil sources, its cumulative environmental impact warrants careful analysis.

Regarding carbon emissions, specifically the carbon footprint, the ILCD 2011 Midpoint+ methodology reports a value of 326,636.40 kg of CO_2_ emitted annually, equivalent to 326.64 metric tons of CO_2_. A very similar result was obtained using the IPCC 2013 GWP 100a method, developed by the Intergovernmental Panel on Climate Change to assess impacts over a 100-year time horizon [46]. According to this methodology, annual emissions were estimated at 333,226.80 kg of CO_2_ (or 333.23 tons of CO_2_).

Although both methodologies, ILCD 2011 Midpoint+ and IPCC GWP 100a, report atmospheric emissions close to 332–333 tonnes of CO_2_ annually, based on the mass and energy data entered into the openLCA software for the EPS densification process, it is important to highlight that the database used, ELCD, contains a wide variety of material and energy flows related to the same processes in the studied system but developed in Europe. This difference in geographic and operational context may influence the results obtained, which led to an alternative calculation of the carbon footprint using the emission factors stipulated by Colombian regulations (Table 1). Under this latter approach, an annual emission of 237.82 tonnes of CO_2_ was determined.

When comparing these results with an alternative scenario where Expanded Polystyrene waste was incinerated instead of being valorized, a significant reduction in carbon dioxide emissions was observed in processes framed within a circular economy. According to the Ecoinvent database, a municipal incineration process of expanded polystyrene generates approximately 3.13 kg of CO_2_ for each kilogram of expanded polystyrene incinerated [47,48]. As detailed in Table 2, under a linear economy scenario, where the life cycle of expanded polystyrene ends with its incineration due to its classification as single-use plastic, annual emissions would substantially increase, reaching an approximate total of 1055.82 tonnes of CO_2_.

According to the results obtained, and in comparison with the standardized methodologies of the IPCC and ILCD, it is determined that the reintegration of Expanded Polystyrene (EPS) waste into new production chains, rather than its final disposal in landfills, where it can take several centuries to decompose [18], or its treatment through incineration processes [46], allows for a significant reduction in carbon dioxide emissions. Specifically, a reduction of up to 729.18 tons of CO_2_ annually can be achieved, which represents a 68.44% decrease in total emissions (Table 3), directly contributing to mitigating the effects of climate change [47].

This reduction in emissions demonstrates that the valorization of waste within a circular economy framework not only reduces the environmental burden associated with waste treatment but also provides an effective solution to combat global warming. By preventing the accumulation of EPS in landfills and its subsequent incineration, greenhouse gas emissions are significantly minimized, thereby improving the sustainability of this plastic materials life cycle.

In addition to the positive contribution of expanded polystyrene (EPS) recycling in reducing the carbon footprint, this process offers additional advantages by allowing the comprehensive recovery of other recyclable materials generated during the EPS cleaning stage. These materials include plastics, PET, cardboard, scrap metal, and strapping, which, when recovered and reintroduced into their respective production chains, enhance the positive impact of recycling and promote the transition towards a circular economy.

The recycling of these materials not only prevents their accumulation in landfills but also contributes to the conservation of natural resources and the reduction in energy demand associated with the production of virgin materials. For example, recycling PET and plastics in general can significantly reduce greenhouse gas emissions, as the production of recycled plastics consumes up to 80% less energy compared to newly produced plastics [46]. Similarly, the valorization of cardboard and scrap metal contributes to the conservation of raw materials such as wood and minerals.

### 3.4. Technical–Economic Evaluation (TEA)

The results obtained from the technical–economic analysis of the densified polystyrene generation process from post-consumer expanded polystyrene (EPS) show that the financial viability, based on an 8-year projection, considering an initial investment made in 2023 and net cash flows from 2024 to 2031, presents a negative Net Present Value (NPV) approximately—US $27,200 with an opportunity rate of 9%. This result indicates that by the end of the 8-year period, the company does not recover the full initial investment, suggesting that the projection period should be extended to achieve financial equilibrium.

On the other hand, the Internal Rate of Return (IRR) of 2.47% begins to indicate that the investment may yield a return, which can be confirmed with the Benefit–Cost (B/C) ratio, where a value of 0.72 was obtained, demonstrating that by year 8, the benefits exceed the costs. However, as mentioned, the investment is still not recovered within this period, thus rejecting the financial viability of the process at this time.

To improve the profitability of the project, an additional scenario was modeled by extending the financial analysis to 12 years. In this new scenario, the NPV becomes positive, reaching a value of US $11,900, demonstrating that by increasing the number of periods, the company will be able to recover its initial investment and generate net benefits. In this regard, the IRR increases to 11.02%, suggesting that the project has greater profitability potential in the long term. Additionally, the Benefit–Cost (B/C) ratio rises to 1.12, indicating that benefits exceed costs from this period onward. These results confirm that the densified EPS production process is financially viable over a 12-year period, allowing the company to recover its initial investment and begin generating net profit.

It is important to highlight that extending the time required to achieve these profits must also consider external factors that could affect the viability of the project, such as fluctuations in raw material costs, environmental policies concerning plastic recycling, and market trends related to the valuation of post-consumer waste. On the other hand, it is important to note that the techno-economic analysis developed is based on operating parameters measured directly at the industrial plant, thus constituting a first deterministic approximation of the economic performance of the process. Detailed information on the TEA, including details of the costs and operating conditions considered, is available in the Supplementary Materials for this study. Given that real data from the operation was available and direct access to the company was provided, no additional sensitivity analyses were performed at this stage of the TEA.

A key factor in enhancing the viability of the project is the incorporation of the Technology Readiness Level (TRL) methodology. This tool is used to assess the progress and technological maturity of a process. The TRLs enable the identification of technological gaps that could improve the efficiency of the production process, as well as provide a framework for designing corrective and preventive actions. This approach helps evaluate both technological and financial risks, fostering the implementation of innovative business models that maximize profitability [49]. Ultimately, combining these tools with detailed financial analyses offers a deeper understanding of the economic feasibility of the post-consumer EPS recycling and valorization process.

## 4. Conclusions

It was found that the overall performance of the studied system regarding material flow was 78.09%, with losses associated with the amount of ordinary waste generated during the cleaning process of recycled Expanded Polystyrene (EPS) and the amount of water used for cooling the finished product blocks (densified polystyrene). On the other hand, the recyclable waste from the EPS post-consumption cleaning process is disposed of by recyclers and other companies for further use, which has a positive environmental impact in the integrated management of waste, especially recyclables. However, the company does not currently valorize the ordinary waste, which is eventually disposed of by another waste management company in landfills. Regarding energy flow, an energy loss of 55.46% and an energy recovery or utilization of 44.54% were determined. These energy losses occur primarily in the raw material reception stage with the intermittent use of the scale, in the EPS cleaning process due to the low efficiency of the motor in both the cutter and the paddle mill, and in the melting and degassing stages due to thermal losses and motor efficiency in the main equipment.

Currently, the company is financially operational; however, the Techno-Economic Analysis demonstrates that the studied system will achieve financial viability in 12 years, meaning that the initial investment will be recovered, and its economic benefits will outweigh production costs. One way to reduce this period for achieving financial viability is by increasing the amount of raw material in order to boost production and consequently revenue, or by entering new markets. Nonetheless, although 12 years is a significant period, it is important to consider that the system has a significant and positive environmental return. This was demonstrated through the evaluation of environmental impacts using the ILCD 2011 Midpoint+ and IPCC GW 100-years methods, where an annual carbon footprint of 332–333 tons of CO_2_ was determined, which is 68.44% lower than the carbon footprint generated when EPS is incinerated instead of reused post-consumption. Therefore, although the studied system has both carbon and water footprints, it is considered that the process generates more positive environmental impacts than negative ones, in addition to representing a recycling model to follow, contributing to plastic waste management. Not only does it recycle post-consumption EPS, but it also recycles other waste materials that have value in markets incorporated into the circular economy.

Based on the analysis of material and energy losses, as well as the economic and environmental aspects, the studied system is technically viable and has the tools to continue as a productive activity that is environmentally friendly. This is because there is a mitigation of environmental impacts compared to those generated when there is no integrated management of recyclable and recoverable waste. Additionally, it is financially viable in the long term, with a dynamic outlook that depends on the economic strategies implemented within the company.

## Figures and Tables

**Table 1:** Carbon Footprint Associated with the Production of High-Density Polystyrene.

Resource	Environmental Burden	Units	Emission Factor (EF)	Unit (EF)	Annual Emissions	Carbon Footprint kg CO_2_	Carbon Footprint Ton CO_2_
Recycled expanded polystyrene	28,110	Kg	0.672	kgCO_2_/kg	226,679.04	237,823.30	237.82
Energy	7370.54	KWh	0.126	kgCO_2_/kWh	11,144.25		
Water	0.001737	Kg	0.083576	kgCO_2_/m^3^	0.0017420		

**Table 2:** Emissions—Incineration of municipal EPS.

Linear Economy—Incineration				
Residue	Monthly Amount (kg)	Monthly Atmospheric Emissions kgCO_2_eq	Annual Atmospheric Emissions kgCO_2_eq	Annual Atmospheric Emissions Ton CO_2_eq
Expanded polystyrene	28,110	87,984.3	1,055,811.6	1055.81

**Table 3:** Percentage reduction in carbon footprint in the production of densified polystyrene from recycled EPS.

Carbon Footprint—IPCC GWP 100a Ton CO_2_	333.23
Carbon Footprint—ILCD 2011 Midpoint+ Ton CO_2_	326.64
Carbon Footprint—Incineration of Post-Consumer EPS (Expanded Polystyrene) Ton CO_2_	1055.81
Reduction in Carbon Footprint (Ton CO_2_)	729.18
% Reduction in Carbon Footprint	68.44

## Data Availability

The original contributions presented in this study are included in the article/Supplementary Materials. Further inquiries can be directed to the corresponding author.

## Supplementary Materials

The following supporting information can be downloaded at: https://www.mdpi.com/article/10.3390/polym18010034/s1. Table S1: Initial Investment—Estimated for 12 months; Table S2: Total annual income (USD); Table S3: Interest and amortization; Table S4: Cash flow.
