# Supplementary material for: Gate-to-Gate Life Cycle Study and Techno-Economic Analysis of an Industrial Process for Producing Densified Polystyrene from Recycled Expanded Polystyrene
Source: Polymers (Basel). 2025 Dec 23;18(1):34. doi: 10.3390/polym18010034 (PMC12787432; doi:10.3390/polym18010034)
Supplement: Supplementary file 1 [file polymers-18-00034-s001.zip › polymers-3992602-supplementary.pdf]

*Supplementary Materials*

*Article*

# Gate-to-Gate Life Cycle Study and Techno-Economic Analysis of an Industrial Process for Producing Densified Polystyrene from Recycled Expanded Polystyrene.

**Eliana Berrio - Mesa [1], Alba N. Ardila A.[1], Erasmo Arriola – Villaseñor [1] and Santiago A. Bedoya – B. [1,*]**

[1] Research Group on Environmental Catalysis and Renewable Energies (CAMER), Faculty of Sciences and Education, Politécnico Colombiano Jaime Isaza Cadavid, Medellín PC 050022, Colombia; eliana_berrio27121@elpoli.edu.co(E.B.M); anardila@elpoli.edu.co (A.N.A.A); erasmoarriola@elpoli.edu.co (E.A.V.); santiago_bedoya27081@elpoli.edu.co (S.A.B.B)
*Correspondence: anardila@elpoli.edu.co

**Table S1.** Initial Investment—Estimated for 12 months.

| DESCRIPTION | COST (USD) |
|---|---|
| Equipment | $60.223,98 |
| Electricity and Water (Utilities) | $19.044,04 |
| Labor | $37.061,77 |
| Land (Lease) | $15.000,00 |
| Raw Materials | $549 |
| Forklift (2.0 ton Capacity) | $899 |
| Purchases and Repairs | $3.834 |
| Additional Expenses | $23.452 |
| Transportation | $32.551,12 |
| Total Initial Investment | $192.615,45 |

**Table S2.** Total annual income (USD).

| Material | Total production (kg) | Cost (USD/kg) | Total monthly revenue (USD) | Total annual revenue (USD) |
|---|---|---|---|---|
| Densified polystyrene | 24644 | $0,70 | $17.173,82 | $206.085,88 |
| Recyclable waste—plastic | 440 | $0,25 | $108 | $1.299,69 |
| Recyclable waste—PET | 202 | $0,10 | $21 | $252,97 |
| Recyclable waste—cardboard | 467 | $0,12 | $57 | $679,66 |
| Recyclable waste—scrap metal | 135 | $0,21 | $28 | $339,37 |
| Recyclable waste—lashing strap | 50 | $0,13 | $6 | $76,92 |
| **TOTAL REVENUE (USD)** | | | | $208.734,50 |

**Table S3.** Interest and amortization.

| **For a period of 8 years (USD)** | | | | |
|---|---|---|---|---|
| **Period** | **Fee** | **Interest** | **Amortization** | **Ending balance** |
| - | - | - | - | $ 96.308 |
| 1 | $ 19.387 | $ 11.557 | $ 7.830 | $ 88.478 |
| 2 | $ 19.387 | $ 10.617 | $ 8.770 | $ 79.708 |
| 3 | $ 19.387 | $ 9.565 | $ 9.822 | $ 69.886 |
| 4 | $ 19.387 | $ 8.386 | $ 11.001 | $ 58.885 |
| 5 | $ 19.387 | $ 7.066 | $ 12.321 | $ 46.564 |
| 6 | $ 19.387 | $ 5.588 | $ 13.799 | $ 32.765 |
| 7 | $ 19.387 | $ 3.932 | $ 15.455 | $ 17.310 |
| 8 | $ 19.387 | $ 2.077 | $ 17.310 | $ 0 |
| **For a period of 12 years (USD)** | | | | |
| **Period** | **Fee** | **Interest** | **Amortization** | **Ending balance** |
| - | - | - | - | $ 96.308 |
| 1 | $ 15.548 | $ 11.557 | $ 3.991 | $ 92.317 |
| 2 | $ 15.548 | $ 11.078 | $ 4.470 | $ 87.847 |
| 3 | $ 15.548 | $ 10.542 | $ 5.006 | $ 82.842 |
| 4 | $ 15.548 | $ 9.941 | $ 5.607 | $ 77.235 |
| 5 | $ 15.548 | $ 9.268 | $ 6.279 | $ 70.956 |
| 6 | $ 15.548 | $ 8.515 | $ 7.033 | $ 63.923 |
| 7 | $ 15.548 | $ 7.671 | $ 7.877 | $ 56.046 |
| 8 | $ 15.548 | $ 6.725 | $ 8.822 | $ 47.224 |
| 9 | $ 15.548 | $ 5.667 | $ 9.881 | $ 37.343 |
| 10 | $ 15.548 | $ 4.481 | $ 11.066 | $ 26.276 |
| 11 | $ 15.548 | $ 3.153 | $ 12.394 | $ 13.882 |
| 12 | $ 15.548 | $ 1.666 | $ 13.882 | $ 0 |

**Table S4.** Cash flow.

**For an 8-year period (USD)**

| | **2023** | **2024** | **2025** | **2026** | **2027** | **2028** | **2029** | **2030** | **2031** |
|---|---|---|---|---|---|---|---|---|---|
| Operating Income | | $ 208.734 | $ 217.710 | $ 227.072 | $ 236.836 | $ 247.020 | $ 257.641 | $ 268.720 | $ 280.275 |
| Financial Income | | | | | $ - | $ - | $ - | $ - | $ - |
| Operating Costs | | $ 177.615 | $ 186.141 | $ 195.076 | $ 204.439 | $ 214.252 | $ 224.537 | $ 235.314 | $ 246.609 |
| Interests | | $ 11.557 | $ 10.617 | $ 9.565 | $ 8.386 | $ 7.066 | $ 5.588 | $ 3.932 | $ 2.077 |
| Depreciation | | $ 12.045 | $ 12.045 | $ 12.045 | $ 12.045 | $ 12.045 | $ 12.045 | $ 12.045 | $ 12.045 |
| **NET TAXABLE INCOME** | | $ 7.517 | $ 8.907 | $ 10.386 | $ 11.965 | $ 13.656 | $ 15.472 | $ 17.429 | $ 19.544 |
| Direct taxes | | $ 2.406 | $ 2.850 | $ 3.324 | $ 3.829 | $ 4.370 | $ 4.951 | $ 5.577 | $ 6.254 |
| Salvage Value (Sale of Assets) | | | | | | | | | $ 60.224 |
| Occasional gain on assets sold (40%) | | | | | | | | | $ 24.090 |
| Non-taxable income | | | | | | | | | |
| Non-deductible operating costs | | | | | | | | | |
| Book value of assets sold (Non-taxable income) | | | | | | | | | |
| **NET INCOME** | | $ 5.112 | $ 6.057 | $ 7.063 | $ 8.136 | $ 9.286 | $ 10.521 | $ 11.852 | $ 49.424 |
| Depreciation | | $ 12.045 | $ 12.045 | $ 12.045 | $ 12.045 | $ 12.045 | $ 12.045 | $ 12.045 | $ 12.045 |
| Salvage value, unsold assets | | | | | | | | | |
| Financial Investments | | | | $ - | | | | | |
| Investment costs | -$ 192.615 | | | | | | | | |
| Loans received | $ 96.308 | | | | | | | | |
| Loan amortization | | $ 7.830 | $ 8.770 | $ 9.822 | $ 11.001 | $ 12.321 | $ 13.799 | $ 15.455 | $ 17.310 |
| **NET CASH FLOW** | -$ 96.308 | $ 9.326 | $ 9.332 | $ 9.285 | $ 9.180 | $ 9.010 | $ 8.767 | $ 8.441 | $ 44.159 |

## For a 12-year period (USD)

| | | 2023 | 2024 | 2025 | 2026 | 2027 | 2028 | 2029 | 2030 | 2031 | 2032 | 2033 | 2034 | 2035 |
|---|---|---|---|---|---|---|---|---|---|---|---|---|---|---|
| + | Operating Income | | $ 208.734 | $ 217.710 | $ 227.072 | $ 236.836 | $ 247.020 | $ 257.641 | $ 268.720 | $ 280.275 | $ 292.327 | $ 304.897 | $ 318.007 | $ 331.682 |
| + | Financial Income | | | | | $ - | $ - | $ - | $ - | $ - | $ - | $ - | $ - | $ - |
| - | Operating Costs | | $ 177.615 | $ 186.141 | $ 195.076 | $ 204.439 | $ 214.252 | $ 224.537 | $ 235.314 | $ 246.609 | $ 258.447 | $ 270.852 | $ 283.853 | $ 297.478 |
| - | Interests | | $ 11.557 | $ 11.078 | $ 10.542 | $ 9.941 | $ 9.268 | $ 8.515 | $ 7.671 | $ 6.725 | $ 5.667 | $ 4.481 | $ 3.153 | $ 1.666 |
| - | Depreciation | | $ 12.045 | $ 12.045 | $ 12.045 | $ 12.045 | $ 12.045 | $ 12.045 | $ 12.045 | $ 12.045 | $ 12.045 | $ 12.045 | $ 12.045 | $ 12.045 |
| = | **NET TAXABLE INCOME** | | $ 7.517 | $ 8.446 | $ 9.409 | $ 10.411 | $ 11.454 | $ 12.545 | $ 13.690 | $ 14.895 | $ 16.169 | $ 17.519 | $ 18.956 | $ 20.493 |
| - | Direct taxes | | $ 2.406 | $ 2.703 | $ 3.011 | $ 3.331 | $ 3.665 | $ 4.015 | $ 4.381 | $ 4.766 | $ 5.174 | $ 5.606 | $ 6.066 | $ 6.558 |
| + | Salvage Value (Sale of Assets) | | | | | | | | | | | | | $ 60.224 |
| - | Occasional gain on assets sold (40%) | | | | | | | | | | | | | $ 24.090 |
| + | Non-taxable income | | | | | | | | | | | | | |
| - | Non-deductible operating costs | | | | | | | | | | | | | |
| + | Book value of assets sold (Non-taxable income) | | | | | | | | | | | | | |
| = | **NET INCOME** | | $ 5.112 | $ 5.743 | $ 6.398 | $ 7.079 | $ 7.789 | $ 8.531 | $ 9.309 | $ 10.129 | $ 10.995 | $ 11.913 | $ 12.890 | $ 50.070 |
| + | Depreciation | | $ 12.045 | $ 12.045 | $ 12.045 | $ 12.045 | $ 12.045 | $ 12.045 | $ 12.045 | $ 12.045 | $ 12.045 | $ 12.045 | $ 12.045 | $ 12.045 |
| + | Salvage value, unsold assets | | | | | | | | | | | | | |
| - | Financial Investments | | | | | | | | | | | | | |
| - | Investment costs | -$ 192.615 | | | | | | | | | | | | |
| + | Loans received | $ 96.308 | | | | | | | | | | | | |
| - | Loan amortization | | $ 3.991 | $ 4.470 | $ 5.006 | $ 5.607 | $ 6.279 | $ 7.033 | $ 7.877 | $ 8.822 | $ 9.881 | $ 11.066 | $ 12.394 | $ 13.882 |
| = | **NET CASH FLOW** | -$ 96.308 | $ 13.166 | $ 13.319 | $ 13.437 | $ 13.517 | $ 13.554 | $ 13.543 | $ 13.477 | $ 13.351 | $ 13.159 | $ 12.891 | $ 12.541 | $ 48.233 |
